# Elevated Intracranial Pressure and Cerebral Edema following Permanent MCA Occlusion in an Ovine Model

**DOI:** 10.1371/journal.pone.0130512

**Published:** 2015-06-29

**Authors:** Adam J. Wells, Robert Vink, Stephen C. Helps, Steven J. Knox, Peter C. Blumbergs, Renée J. Turner

**Affiliations:** 1 Adelaide Centre for Neuroscience Research, School of Medical Sciences, University of Adelaide, Adelaide, South Australia, 5005, Australia; 2 Department of Neurosurgery, Royal Adelaide Hospital, Adelaide, South Australia, 5000, Australia; 3 Faculty of Health Sciences, University of South Australia, Adelaide, South Australia, 5000, Australia; 4 Department of Radiology, Royal Adelaide Hospital, Adelaide, South Australia, 5000, Australia; 5 Tissue Pathology, South Australia Pathology, Adelaide, South, Australia, 5000, Australia; Indian Institute of Integrative Medicine, INDIA

## Abstract

**Introduction:**

Malignant middle cerebral artery (MCA) stroke has a disproportionately high mortality due to the rapid development of refractory space-occupying cerebral edema. Animal models are essential in developing successful anti-edema therapies; however to date poor clinical translation has been associated with the predominately used rodent models. As such, large animal gyrencephalic models of stroke are urgently needed. The aim of the study was to characterize the intracranial pressure (ICP) response to MCA occlusion in our recently developed ovine stroke model.

**Materials and Methods:**

30 adult female Merino sheep (n = 8–12/gp) were randomized to sham surgery, temporary or permanent proximal MCA occlusion. ICP and brain tissue oxygen were monitored for 24 hours under general anesthesia. MRI, infarct volume with triphenyltetrazolium chloride (TTC) staining and histology were performed.

**Results:**

No increase in ICP, radiological evidence of ischemia within the MCA territory but without space-occupying edema, and TTC infarct volumes of 7.9+/-5.1% were seen with temporary MCAO. Permanent MCAO resulted in significantly elevated ICP, accompanied by 30% mortality, radiological evidence of space-occupying cerebral edema and TTC infarct volumes of 27.4+/-6.4%.

**Conclusions:**

Permanent proximal MCAO in the sheep results in space-occupying cerebral edema, raised ICP and mortality similar to human malignant MCA stroke. This animal model may prove useful for pre-clinical testing of anti-edema therapies that have shown promise in rodent studies.

## Introduction

Malignant middle cerebral artery (MCA) stroke is associated with rapid neurological deterioration secondary to space-occupying cerebral edema following large volume MCA territory infarction [1]. It accounts for 10–15% of all supratentorial strokes but a disproportionately high 30-day mortality rate of 60–80% [2, 3], compared with approximately 20% for all ischemic strokes [4]. Death results from rapid edema formation, raised intracranial pressure (ICP) and transtentorial herniation, typically within 2–5 days of stroke onset, but often as early as 24 hours [1, 3, 5]. Despite this, current medical intervention for cerebral edema is limited to the use of osmotic diuretics or invasive surgery with decompressive craniectomy [5]. Pharmacotherapies such as mannitol and hypertonic saline aim to dehydrate the swollen brain by drawing out excess fluid into the vasculature [5]. Overall, these current non-surgical therapies are ineffective or inadequate [5]. Decompressive craniectomy is a powerful surgical tool whereby a flap of bone is removed overlying the swollen brain tissue to provide room for the expansion of the swollen brain and reduce dangerously elevated ICP. Although decompressive craniectomy significantly reduces the number of dead or severely disabled patients following malignant MCA stroke [6], it is also a highly invasive procedure and not without its own morbidity and mortality [7]. As such, alternate non-surgical treatments for cerebral edema and elevated ICP are urgently required. However, to date there has been very poor translation of therapeutics from experimental models to the clinical setting to date [8], clearly this disconnect needs to be addressed and alternate approaches are urgently required to improve outcome and survival for stroke patients. More rigorous pre-clinical testing is likely to facilitate this outcome.

A number of factors may have attributed to the lack of clinical translation of experimental therapeutics, one of which is the choice of animal model. Rodent models of MCA occlusion (MCAO) are widely used, in particular the intraluminal thread model [9]. Our understanding of the pathophysiological mechanisms of cerebral ischemia at a cellular and molecular level has increased considerably due to knowledge gained from such rodent stroke models[10]. However, given the gross neuroanatomical differences between small animal lissencephalic brains and large animal/human gyrencephalic brains, such small animal models do not accurately reproduce the intracranial pathophysiology which occurs after large cerebral insults in humans [11]. This is particularly relevant in the context of space-occupying lesions and raised ICP, in which the rodent brain is frequently associated with an inconsistent ICP response and low/variable mortality despite very large insults [11–13].

An ovine model of gyrencephalic MCAO has previously been characterized, including descriptions of magnetic resonance imaging (MRI), positron emission tomography, behavioral phenotyping and histopathology after permanent occlusion of one branch, two branches or the main MCA trunk [14]. More recently, we have described the surgical approach to sheep proximal MCA occlusion, demonstrating almost complete MCA territory ischemia following permanent proximal MCAO and smaller lesions with transient occlusion and reperfusion [15]. We observed histological evidence of large areas of ischemia, blood-brain barrier (BBB) dysfunction and early edema development but no elevation in ICP or radiological evidence of space-occupying edema or cerebral herniation and this was due to the short 4 hour time period examined. We propose that in this large animal model of stroke, a longer monitoring period may reproduce the intracranial pathophysiological changes observed in human MCA territory stroke such as elevated intracranial pressure and herniation syndromes. As such, the aim of the current study was to fully characterize the ICP (primary objective), radiological and histopathological (secondary objectives) response to permanent and transient ovine proximal MCAO at 24 hours after stroke onset. In this paper we demonstrate that this model is a good candidate for screening of potential therapeutics prior to clinical assessment.

## Experimental Procedure

### Ethics Statement

All studies were approved by the Animal Ethics Committees of the University of Adelaide (Approval M-2010-125) and SA Pathology (Approval #44/10). All experiments were conducted in accordance with the Australian National Health and Medical Research Council code of practice for the care and use of animals for scientific purposes (8^th^ edition, 2013). Animals remained under general anesthesia for the entire duration of the experiment and did not experience any pain or distress.

### Animals and study design

30 adult female naïve wild-type *Merino sheep* 18–36 months old (mean: 59.5 +/- 7.3kg; range: 45–73kg) were allocated to the study. Animals were group housed in the conventional sheep paddocks at the Large Animal Research Facility (Gilles Plains, South Australia), with free access to food, water and shelter. All animals were fasted overnight before the day of surgery. Animals were intra-operatively randomized to one of three groups: permanent right proximal MCAO (n = 10), 2 hours of temporary aneurysm clip right proximal MCAO followed by reperfusion (n = 12) or sham surgery (n = 8). Randomization was achieved by selection a pre-sealed envelope (from a shuffled pile) which contained a surgery allocation card listing either sham, permanent MCAO or transient MCAO. Animals underwent sham surgery or proximal MCAO and were then monitored under general anesthesia for a 24 hour period for ICP (primary outcome measure) and brain tissue oxygenation, followed by, infarct volume staining and histological assessment at the end of the monitoring period. Note that only a subset of animals underwent MRI (permanent MCAO n = 6; temporary MCAO n = 6). The primary outcome measured was intracranial pressure and the secondary outcomes measured were MRI characterisitics, lesion volume and histological assessment.

### Anesthesia

Anesthesia was induced with intravenous thiopentone (1000mg in 20mls, Jurox Pty Ltd, Australia), animals were then intubated and maintained on inhalational isoflurane (1.5–3%; Veterinary Companies of Australia) in a mixture of oxygen and room air, plus intravenous ketamine infusion (4.0mg/kg/hour; Parnell Australia Pty Ltd, via a femoral line. Animals were maintained under general anesthesia for the entire duration of the experiment (~30 hours), as this was a requirement of institutional ethical approval.

### Physiological Monitoring

An arterial catheter was placed in the right femoral artery for continuous blood pressure monitoring and periodic arterial blood gas sampling. With the animal in the sphinx position, burr holes were placed symmetrically and a left hemisphere Codman ICP monitor (Codman & Shurtleff Inc., MA) and right hemisphere brain tissue oxygen (PbtO_2_) LICOX probe (Integra LifeSciences, NJ) were inserted, as previously described in detail [15]. In addition to the established protocol, intramuscular antibiotics (Cephalexin 15mg/kg, Virbac Pty Ltd, Australia) were administered at induction and 12 hourly until the end of the experiment, an indwelling urinary catheter was inserted for urine collection and output measurement, and intravenous fluids were administered as an infusion via the femoral venous catheter (Hartmann’s solution 4mL/kg/h, Baxter Pty Ltd, Australia). To maintain a neutral acid-base status, the strongly alkaline saliva drool was collected and periodically returned via an orogastric feeding tube, and sodium bicarbonate 8.4% (Pfizer Australia Pty Ltd, Australia) was added to the intravenous infusion as required. Core body temperature was monitored and normothermia was maintained with a heating pad and blankets. Animals were monitored under general anesthesia as previously described [15] for 24 hours after MCAO or sham surgery and their mean arterial blood pressure, ICP, brain tissue oxygenation recorded. Periodic blood analysis was conducted to confirm physiological pH and arterial oxygen and carbon dioxide partial pressures, with ventilation parameters adjusted as required. Such physiological monitoring was initiated following induction of general anesthesia and was continued for a 24hour period following stroke or sham surgery induction.

### Surgical Approach to Proximal Middle Cerebral Artery Occlusion

We have previously described the surgical approach to proximal MCA occlusion in detail [15]. Briefly, a 50mm vertical incision was made behind the right eye, terminating at the zygomatic arch. The underlying muscle was retracted, coronoid process removed and underlying skull revealed. A small craniotomy was performed at the junction of the parietal and squamous temporal bones with a high-speed pneumatic drill using a 5mm cutting burr (Midas Rex, Medtronic, MN). A horse-shoe shaped durotomy was performed with an inferiorly based flap. All intra-dural work was carried out with loupe magnification and a head mounted light source (Surgical Acuity, WI). The proximal MCA was location by gentle upwards retraction of the anterior temporal lobe. Animals then underwent either 1) sham surgery, in which the proximal MCA was dissected but not occluded; 2) permanent occlusion in which the proximal MCA was occluded via Malis bipolar diathermy forceps (ValleyLab Inc, CO); or 3) temporary occlusion with reperfusion, in which the proximal MCA was occluded with application of a Sugita temporary mini straight aneurysm clip (Mizuho Medical Inc, Japan) which was removed 2h later. The exposed brain was irrigated with saline during surgery to prevent drying out of the cerebral cortex, particularly in temporary occlusion animals where there was a 2h delay between aneurysm clip application and wound closure. After the completion of sham or MCA occlusion surgery the dura was approximated and closed watertight with ethyl cyanoacrylate (Bostik, Australia) and reinforced with dental acrylic cement (Lang Dental, IL) that was manipulated into the edge of the craniotomy, maintaining the shape of the cranial cavity and importantly, the homeostasis of ICP dynamics. The wound was closed in layers and the head was then returned to a neutral position for monitoring under general anesthesia.

### Magnetic Resonance Imaging

At 24 hours following stroke onset, a subgroup of animals (permanent MCAO n = 6, temporary MCAO n = 6) underwent imaging in a 1.5T Siemens Sonata MRI scanner (Siemens AG, Munich, Germany) using the protocol previously described [15]. Radiological infarct volume was calculated as a percentage of whole brain on coronal section using the diffusion weighted images (DWI), corrected for edema using a modified Swanson calculation [16]. Cerebral edema was calculated on coronal T2 weighted imaging sequences (T2WI) as a percentage of whole brain uncorrected. Midline shift was assessed on axial T1 weighted imaging sequences (T1WI), and measured in millimeters at the level of the interventricular foramen. Cerebral herniation and brainstem compression was identified on axial and sagittal T1WI.

### Histological Examination and Infarct Volume Assessment

At the end of the 24 hour monitoring period, or after MRI, animals were administered intravenous heparin (5000I.U./5ml; Pfizer, NY) and killed via common carotid perfusion fixation with cold TRIS-buffered saline under Isoflurane anesthesia. The brains were subsequently rapidly removed, sliced into 10mm coronal slices using a custom made matrix, and 2,3,5-triphenyltetrazolium chloride (TTC; Sigma-Aldrich Pty Ltd, Australia) staining performed to determine infarct volume, as previously described in rodent stroke [17]. On the TTC stain, non-infarcted tissue stains red/pink in color and infarcted tissue remains a pale cream/white color. Brain slices were incubated in 3% TTC at 37°C under dark room conditions for 20 min, turning once. Anterior and posterior sides of all brain slices were photographed on a flatbed scanner (Canon CanoScan LiDE700F, Canon Inc., Japan). The degree of infarction was determined by an observer blinded to the surgery groups and experienced in the evaluation of infarct determination, with infarct volume calculated as a percentage of whole brain corrected for edema via the modified Swanson calculation [16]. Sections were then immersion fixed in 10% neutral-buffered formalin for a minimum of 7 days prior to being processed for histological examination by H&E, albumin (dilution 1:2000, Dako, Glostrup, Denmark) immunohistochemistry, and Weil’s stain (myelin degeneration). TUNEL assays were performed using a modified technique of the method of Portera-Cailliau et al. [18] (TdT 0.15 U/μl; Promega, Madison, WI; and biotin-16-dUTP, 10 μM; Roche, Castle Hill, Australia).

### Statistical Analysis

Data analysis was conducted by an individual blinded to the surgery group allocation (sham, temporary MCAO and permanent MCAO). All data is expressed as mean +/- standard deviation, with the exception of the ICP and PbtO_2_ results, which were not normally distributed and did not have uniform variance. Raw ICP and PbtO_2_ raw data underwent a logarithmic exponential transformation and were then expressed as geometric mean with standard deviation [19]. Physiological data (arterial blood pressure, ICP, PbtO_2_, PaO_2_, PaCO_2_, core body temperature) was analyzed using two-way analysis of variance followed by individual Bonferroni tests (Prism Version 5.0d, Graphpad, CA). Physiological parameters were analyzed pre-MCAO or sham surgery, and at hourly intervals until the completion of the experiment, with the exception of arterial blood gas data that was collected every 4 hours. Lesion volume data, TUNEL area of immunoreactivity/cell counts and MRI characteristics were analyzed by individual student t-tests. A p-value of p<0.05 was considered significant. All animals were included in the analysis.

## Results

### Surgery, Mortality and Basic Physiology

There were no surgical complications and no animals were excluded from the analysis due to technical complications with the procedure. All sham and temporary MCAO animals survived the 24 hour monitoring period. Of the permanent MCAO animals, 3/10 (30%) died prematurely secondary to raised ICP and brainstem compression. Mean arterial blood pressure, PaO_2_, PaCO_2_ and core body temperature were within physiological parameters, on the whole there were no difference between groups throughout the 24 hour monitoring period. Of exception, were those animals which died prematurely, their physiological responses were characterized initially by an elevation in mean ICP and widening of the ICP pulse amplitude, followed by a rise and then fall in end tidal CO_2_, and finally arterial hypotension and cardiac arrest. Mean time to death in animals dying prematurely was 19h39min +/- 2h55min (range: 16h20min–22h30min). One of the non-survivors had MRI examination at the point of arterial hypotension but prior to cardiac arrest. Such mortality is representative of the human condition and therefore a clinically relevant feature of the model. In light of this, the surgical approach to MCAO was not altered in order to reduce this figure.

### Intracranial Pressure

**Fig 1:**
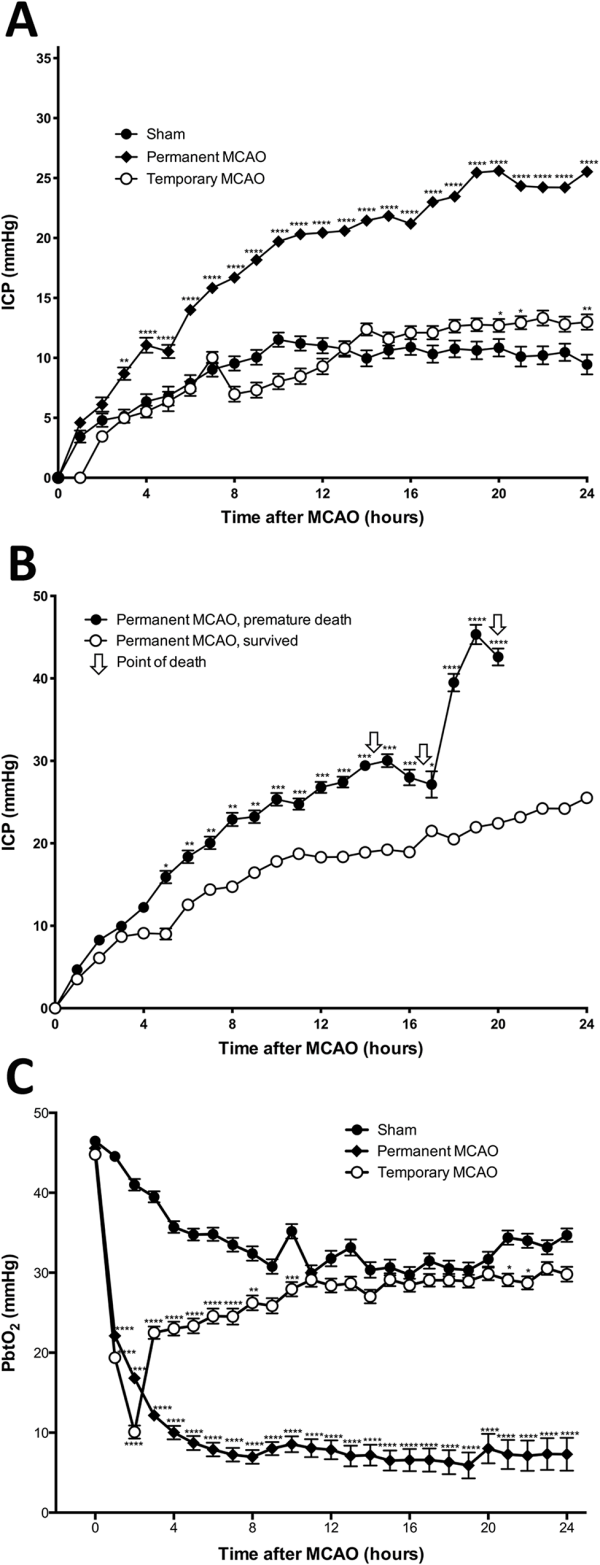
Mean 24 hour ICP and PbtO_2_ following MCAO. Mean ICP following sham surgery, temporary MCAO or permanent MCAO (A). Mean ICP following permanent MCAO, animals that died within the 24-hour monitoring period versus animals that survived (B). Mean PbtO_2_ following temporary and permanent MCAO or sham surgery (C). ICP, intracranial pressure; MCAO, middle cerebral artery occlusion; PbtO_2_, partial pressure of brain tissue oxygen, n = 8–12/gp.

ICP monitoring was conduced in n = 8 shams, n = 12 temporary and n = 10 permanent MCAO animals. The mean pre-operative ICP was 6.4 +/- 1.9mmHg, with no difference between groups (Fig 1A). ICP rose above pre-operative levels in the sham group and was maintained at approximately 10mmHg for the duration of the monitoring period. In temporary MCAO animals, ICP rose slightly higher to 13.0 +/- 2.0mmHg by 24 hours, however with no overall significant difference compared with sham animals. After permanent MCAO, ICP rose significantly compared with sham (p<0.0001), and remained elevated for the duration of the monitoring period with a continuous upward trend. Permanent MCAO animals that died prematurely (Fig 1B) also had a significantly elevated mean ICP compared with permanent MCAO animals that survived (27.0 +/- 1.3mmHg vs. 18.5 +/- 1.3mmHg, p<0.0001), which was sustained for the duration of the monitoring period.

### Brain Tissue Oxygen

PbtO_2_ monitoring was conduced in n = 8 shams, n = 12 temporary and n = 10 permanent MCAO animals. The mean baseline PbtO_2_ for all animals was 45.4 +/- 1.3mmHg, with no difference between groups (Fig 1C). In sham animals, PbtO_2_ showed a gradual decline over the first 8 hours, to eventually plateau at approximately 30–35mmHg for the remainder of the experiment. In temporary occlusion animals baseline PbtO_2_ was 44.8 +/- 1.4mmHg prior to aneurysm clip application, falling to 10.1 +/- 2.3mmHg 2 hours after MCAO, immediately prior to clip removal and reperfusion (p<0.0001). It then rapidly rose to 22.5 +/- 2.2mmHg 1 hour after reperfusion, then gradually climbed to 29.1 +/- 2.5mmHg 9 hours following reperfusion, at which point any difference to sham lost significance. Temporary MCAO PbtO_2_ plateaued at approximately 28–30mmHg for the remainder of the experiment. Permanent occlusion animals demonstrated a baseline of 45.6 +/- 1.4mmHg, which declined significantly following MCAO to a low of 7.0 +/- 2.3mmHg at 8 hours (p<0.0001), and continued to stay low to eventually plateau at approximately 7mmHg by 24 hours (p<0.0001). Permanent MCAO PbtO_2_ remained the lowest of all three groups at the conclusion of the experiment, with temporary MCAO approaching sham levels.

### MRI

**Fig 2:**
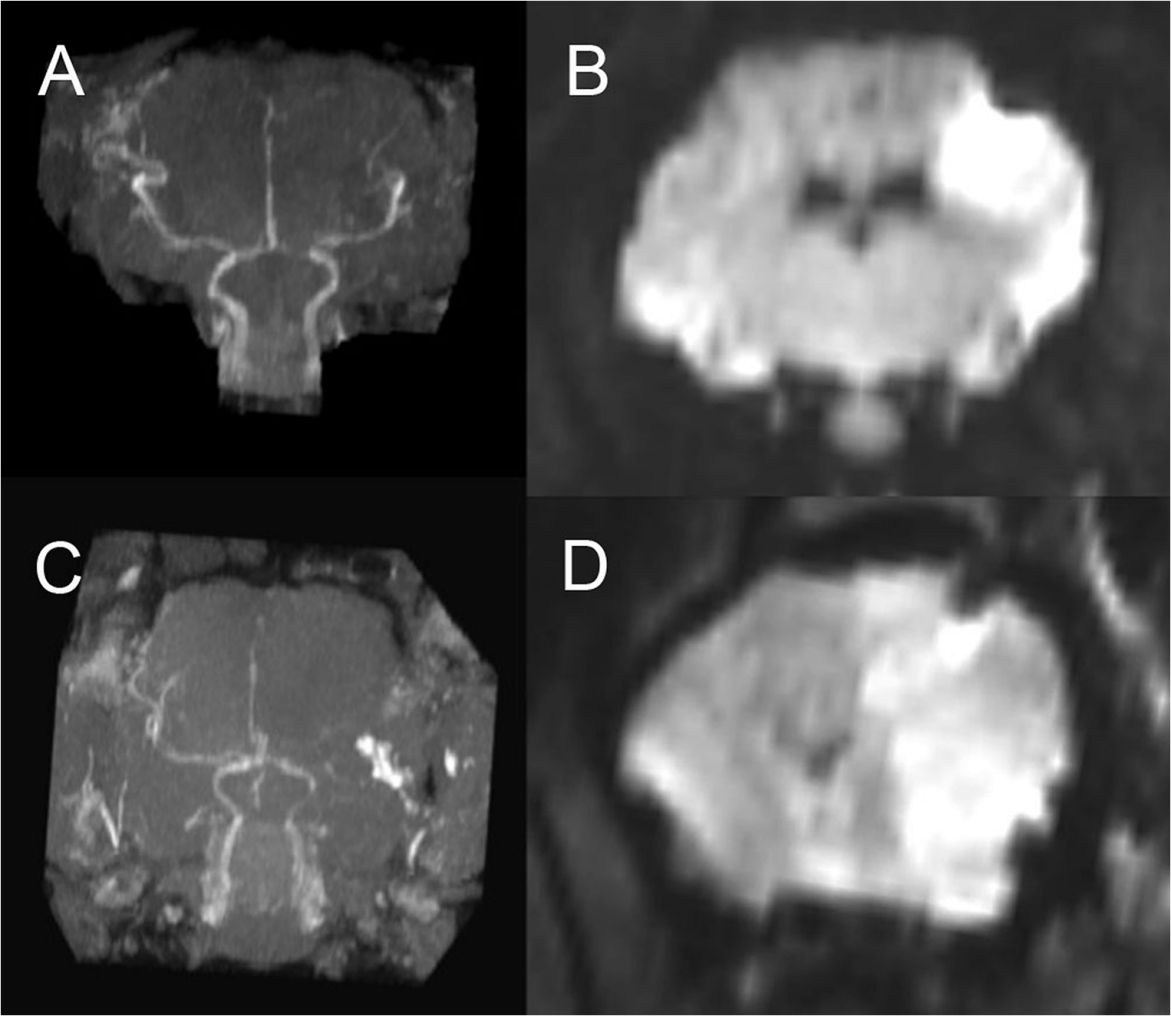
MRI findings at 24 hours following stroke: MRA and DWI. Temporary MCAO MRA demonstrates reperfusion in the right MCA territory (A), and diffusion deficit on DWI in the right MCA cortex (B). Permanent MCAO MRA shows no flow beyond the right proximal MCA (C), and a larger diffusion deficit involving the whole MCA territory including subcortical structures (D). DWI, diffusion weighted imaging; MCA, middle cerebral artery; MCAO, middle cerebral artery occlusion; MRA, magnetic resonance angiography, n = 6/gp.

**Fig 3:**
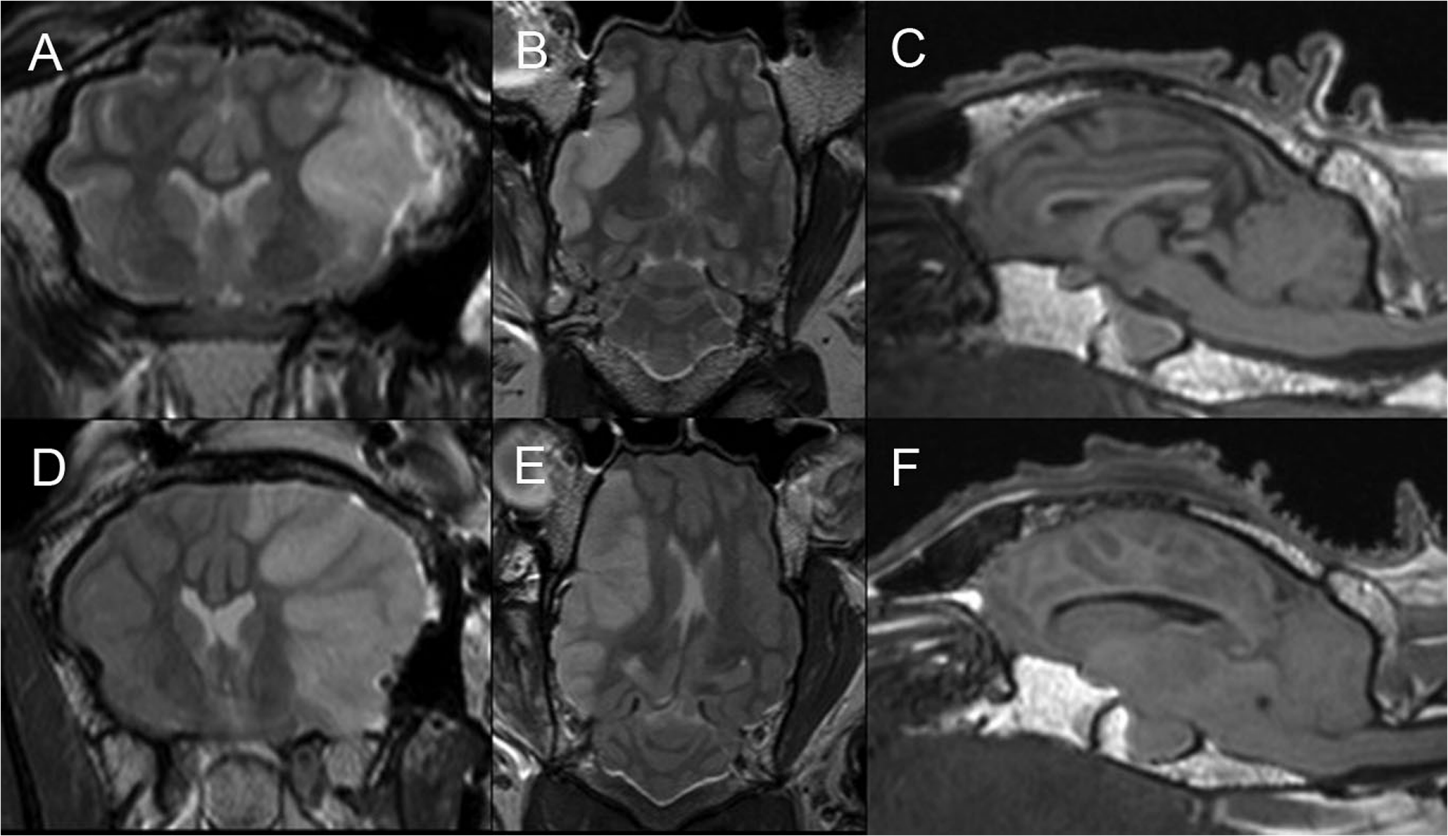
MRI findings at 24 hours following stroke: T1 and T2 weighted imaging. Temporary MCAO demonstrates cerebral edema in a right MCA distribution similar to the diffusion deficit in *Fig 2B* on T2 coronal imaging (A), and no mass effect or midline shift on T2 axial imaging (B). Sagittal T1 sequences show preserved basal cisterns and posterior fossa CSF spaces (C). T2 coronal (D) and axial (E) imaging after permanent MCAO show cerebral edema distributed as for the diffusion deficit in *Fig 2D*, with associated mass effect and midline shift. Sagittal T1 imaging demonstrates effacement of the basal cisterns and cisterna magna, tonsillar herniation and brainstem compression (F). MCA, middle cerebral artery; MCAO, middle cerebral artery occlusion, n = 6/gp.

MRI was performed in 6/10 permanent MCAO and 6/12 temporary MCAO animals; MRI characteristics are shown in Table 1. MRA confirmed no flow beyond the proximal MCA in all permanent MCAO animals (Fig 2C), and reperfusion of the right MCA territory in all temporary MCAO animals (Fig 2A). The mean DWI deficit for permanent MCAO (Fig 2D) animals was 25.4 +/- 6.8%, compared with 10.7 +/- 3.9% for temporary MCAO (Fig 2B) animals (p = 0.001). Cerebral edema measured on coronal T2WI revealed a mean volume of 25.0 +/- 4.9% for permanent MCAO animals, and 5.4 +/- 4.1% for temporary MCAO animals (p<0.0001, Fig 3A and 3D). Mean midline shift was 3.3 +/- 0.6mm for permanent MCAO animals (Fig 3E), compared with 1.0 +/- 0.8mm for temporary MCAO animals (Fig 3B; p = 0.0002). None of the temporary MCAO animals demonstrated transtentorial herniation or brainstem compression (Fig 3C), whereas herniation was present in 3 out of 6 (50%) permanent MCAO animals, seen on MRI as crowding around the foramen magnum, effacement of the cisterna magna and tonsillar herniation (Fig 3F). These animals also demonstrated significant local mass effect with sulcal effacement. Permanent MCAO animals that herniated (n = 3, including 1 animal that died prematurely) demonstrated a mean DWI lesion volume of 27.7 +/- 5.5%, a mean T2WI edema volume of 27.4 +/- 3.6%, and mean midline shift of 3.6 +/- 0.5mm; permanent MCAO animals that did not herniate (n = 3, all survived) demonstrated a mean DWI lesion volume of 23.2 +/- 8.4% (p = 0.479), a mean T2WI edema volume of 22.6 +/- 5.4% (p = 0.269), and mean midline shift of 3.0 +/- 0.7mm (p = 0.298).

**Table 1:** MRI characteristics and TTC infarct volume at 24 hours after MCAO.

Stroke Type			MRI characteristics at 24 hours			
	Animal	TTC infarct volume (%)	DWI volume (%)	T2WI volume (%)	Midline shift (mm)	Herniation (Y/N)
**Permanent MCAO**	1*	30.9	33.8	31.5	3.8	Y
	2	32.8	32.4	26.1	3.1	N
	3	24.9	23.3	24.7	4.1	Y
	4	23.8	21.0	25.3	3.4	N
	5	14.1	16.1	16.3	2.5	N
	6	28.1	25.9	26.0	3.1	Y
	**Mean ±SD**	**25.76 ±6.66**	**25.41 ±6.78**	**24.98 ±4.90**	**3.33 ±0.56**	
**Transient MCAO**	7	2.6	14.7	2.3	0	N
	8	14.1	7.5	3.7	0.9	N
	9	5.8	13.9	5.1	1.6	N
	10	4.8	7.1	1.9	1.6	N
	11	0.9	7.0	5.9	0	N
	12	12.4	14.1	13.2	1.9	N
	**Mean ±SD**	**6.76 ±5.33**	**10.71 ±3.86**	**5.35 ±4.14**	**1 ±0.8**	

*Animal 1 died prematurely at 16h40min. DWI, diffusion weighted imaging; MCAO, middle cerebral artery occlusion; MLS, midline shift; MRI, magnetic resonance imaging; T2WI, T2 weighted imaging; TTC, tetrazolium chloride.

### Infarct Volume

**Fig 4:**
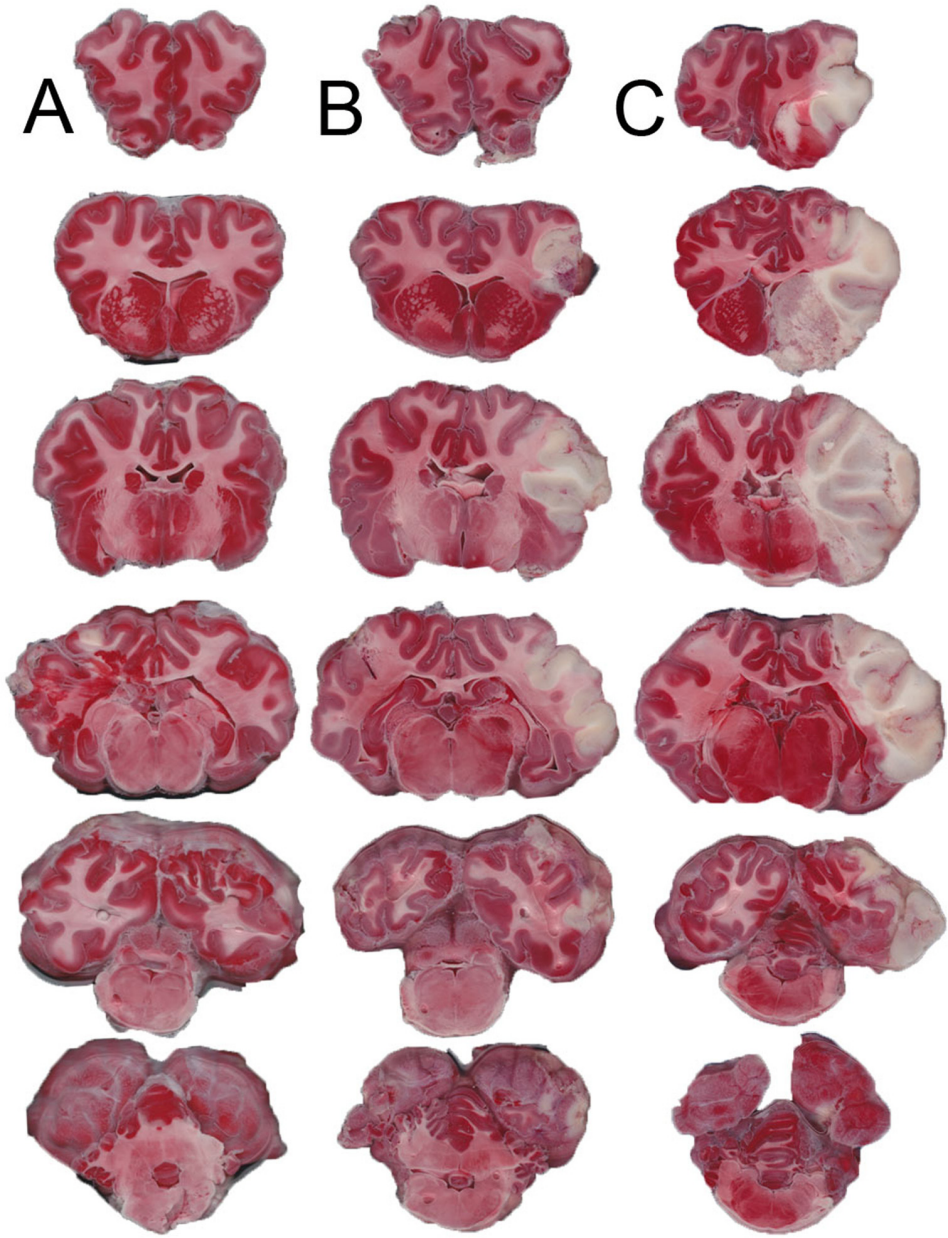
TTC at 24 hours, coronal stack. Unstained brain tissue represents cerebral ischemia. There is no evidence of ischemia in sham animals in the left column (A), small cortical ischemia in temporary MCAO animals in the center column (B), and large MCA territory ischemia in permanent MCAO animals in the right column (C). MCA, middle cerebral artery; MCAO, middle cerebral artery occlusion; TTC, 2,3,5-triphenyltetrazolium chloride, n = 5 shams, n = 11 transient MCAO, n = 8 permanent MCAO.

TTC staining was performed in 5/6 sham animals, 11/12 temporary MCAO animals, and 8/10 permanent MCAO animals. There was no TTC evidence of infarction in any of the sham animals (Fig 4B). All temporary MCAO animals demonstrated TTC pallor within the right MCA cortex (Fig 4A); subcortical structures were involved in 6 of 11 animals, most frequently affecting the head of caudate nucleus. The mean TTC infarct volume for temporary MCAO was 7.9 +/- 5.1%. Permanent MCAO (Fig 4C) animals demonstrated significantly larger TTC infarct volumes that generally affected the whole MCA supplied cortex and typically most of the subcortical structures (caudate, putamen, thalamus), with mean TTC infarct volumes of 27.4 +/- 6.4% (p<0.0001). One permanent MCAO animal demonstrated infarction in the contralateral posterior cerebral artery territory (PCA), attributed to raised ICP, uncal herniation and occlusion of the PCA on its passage through the tentorial notch. This animal died prematurely at 20 hours following MCAO and had a highest mean ICP of 63.1 +/- 7.8mmHg with a pulse amplitude of 24.7mmHg in the hour prior to death. Animals that herniated on MRI tended to have larger TTC infarct volumes than permanent MCAO animals that did not herniate (27.9 +/- 3.0% vs. 23.6 +/- 9.4%, p = 0.485) but this was not statistically significant.

### Histological Assessment

**Fig 5:**
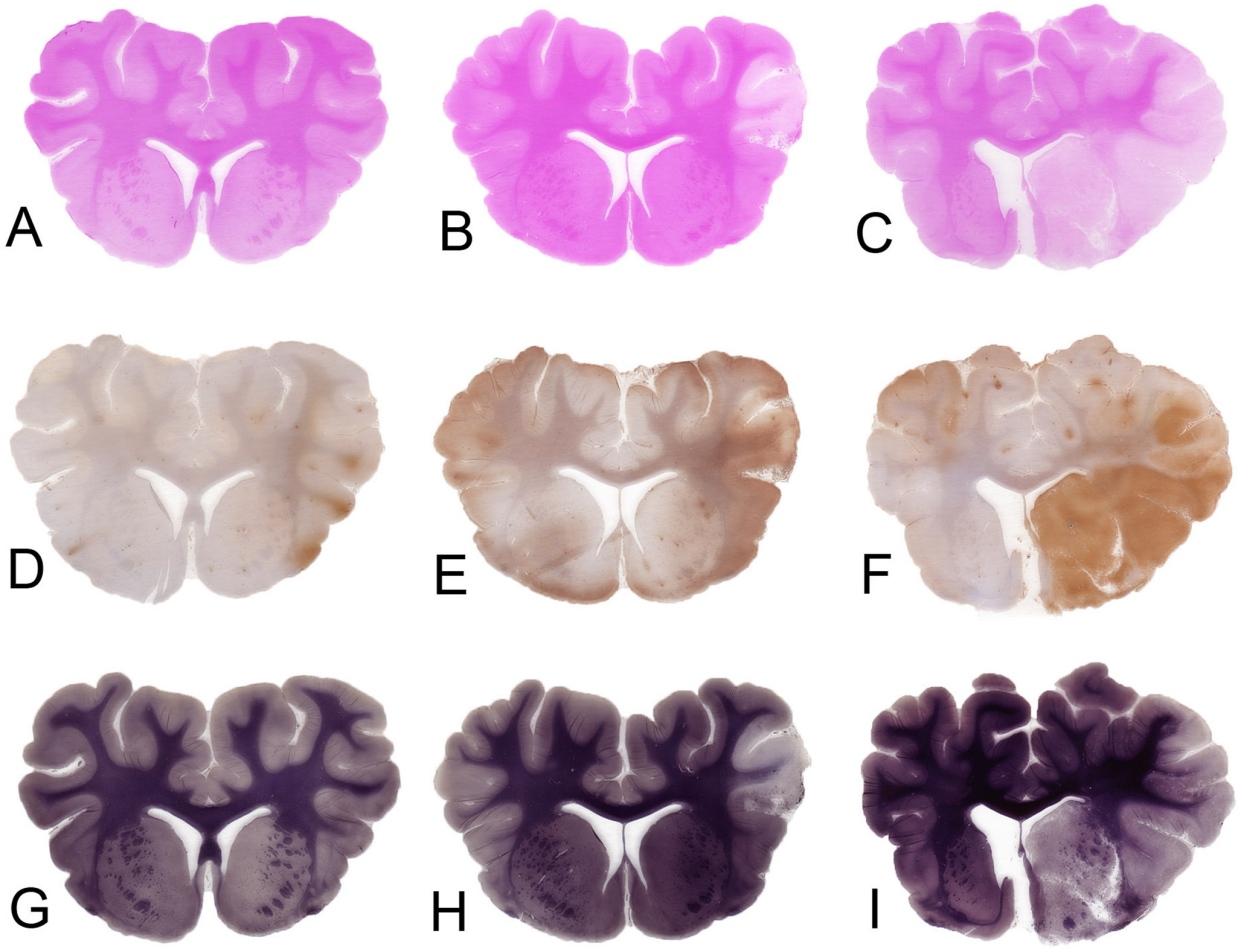
Histopathology, coronal section. Section level with the origin of the MCA. H&E for sham surgery (A), temporary MCAO (B) and permanent MCAO (C). Albumin immunostaining for sham (D), temporary MCAO (E) and permanent MCAO (F). Weil stain for sham (G), temporary MCAO (H) and permanent MCAO (I). MCA, middle cerebral artery; MCAO, middle cerebral artery occlusion, n = 8 shams, n = 12 transient MCAO, n = 10 permanent MCAO.

**Fig 6:**
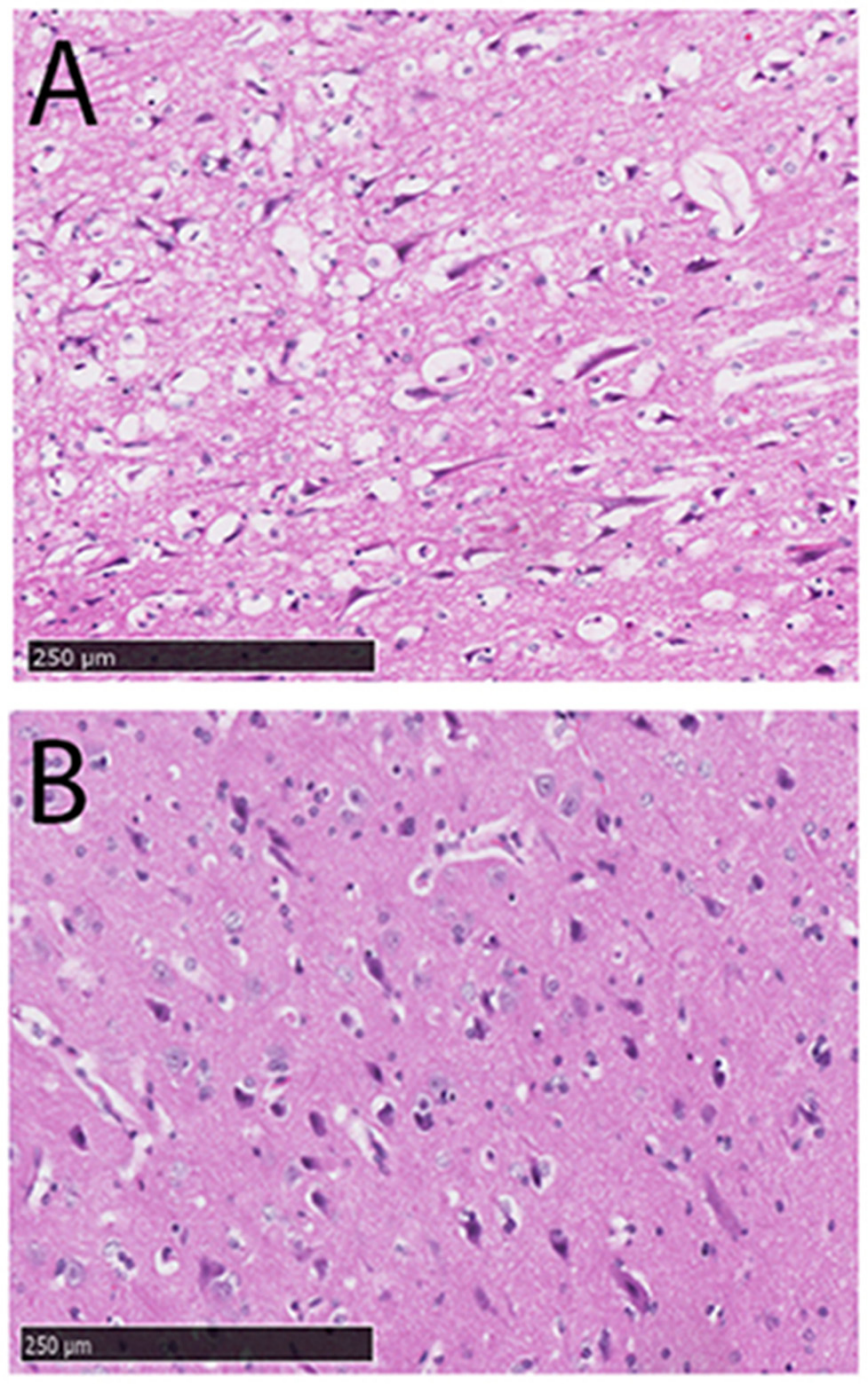
Histopathology for H&E. The infarct was evident as a region of tissue pallor, charcterised by extensive cell injury/death and tissue vacuolation (A). The was selective cell sparing and cell injury/loss within the penumbral tissue (B). n = 8 shams, n = 12 transient MCAO, n = 10 permanent MCAO.

**Fig 7:**
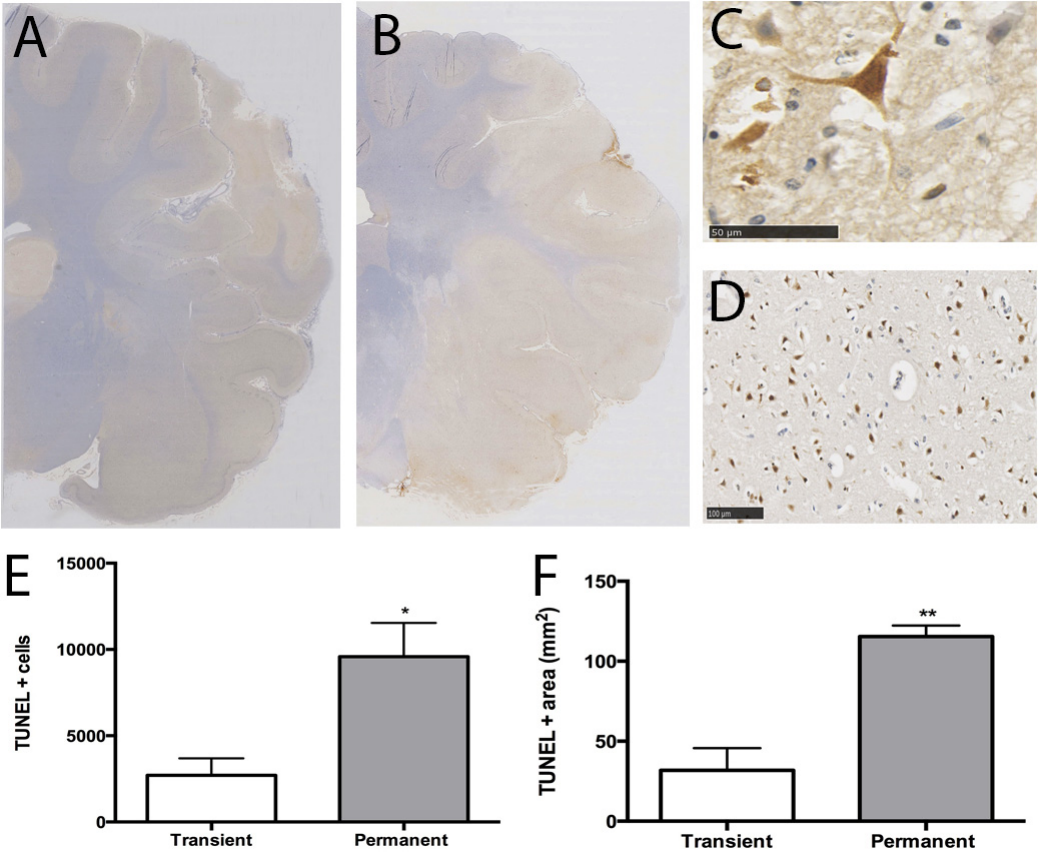
TUNEL staining. TUNEL immunoreactivity, indicative of cell death, was observed throughout the infarcted tissue. The region of TUNEL immunoreactivity was larger and more widespread following permanent (B) than transient MCAO (A). TUNEL positive cells (C) were observed throughout the infarcted tissue, typically seen in areas of tissue vacuolation and extensive cell injury (D). The number of TUNEL positive cells (E) and total area of TUNEL immunoreactivity (F) was significantly higher following permanent than transient MCAO. n = 3/gp.

Histopathological examination revealed different patterns of injury between the two MCAO groups (Figs 5 and 6). With the exception of TUNEL staining (n = 3 temporary MCAO, n = 3 permanent MCAO) all histopathology was conducted in all animals (n = 8 sham, n = 12 temporary MCAO, n = 10 permanent MCAO). Temporary MCAO (Fig 5B) sections stained for H&E demonstrated discrete regions of infarct whereas permanent MCAO (Fig 5C) demonstrated larger infarct corresponding to TTC examination (Fig 4). On closer examination, cells within the infarct core were shrunken, abnormally shaped and vacuolated (Fig 6A), whereas there was greater preservation of tissue architecture and cell integrity in the penumbra (Fig 6B). The Weil stain (Fig 5) was used to show myelin degeneration. Transient MCAO (Fig 5H) resulted in discrete regions of myelin loss, whereas permanent MCAO (Fig 5I) produced large areas of myelin loss, corresponding to the large size of the infarcts seen with TTC staining and on H&E. TUNEL staining, a marker of cell death, showed widespread immunoreactivity within permanent MCAO tissue compared with transient MCAO tissue (Fig 7A and 7B). Indeed, the number of TUNEL positive cells (9588+/-1948 vs. 2702+/- 989; p<0.05) and the total area of positive TUNEL staining (115+/-6.87 vs. 31.86+/-7.99; p<0.01) were both significantly higher following permanent compared to transient MCAO (Fig 7E and 7F), once again in keeping with the infarct volume data. TUNEL positive cells (Fig 7C) were observed throughout the infarct following both transient and permanent MCAO (Fig 7D). Albumin immunohistochemistry was used as a marker of BBB disruption and a surrogate marker of vasogenic edema. Small regions of albumin immunoreactivity were apparent in transient MCAO tissue, compared with large regions in the permanent MCAO tissue (Fig 5D, 5E and 5F) and corresponded well with MRI T2WI evidence of edema (Fig 3A and 3D).

## Discussion

Decompressive craniectomy for malignant MCA stroke improves early survival and outcome by preventing brainstem compression secondary to transtentorial herniation [7]. Novel therapies limiting BBB permeability [20] and vasogenic edema [21, 22] after rodent ischemic stroke have proven to be as effective as or superior to surgical decompression. However, the marked differences between rodent and human intracranial anatomy, particularly the delicate rodent tentorium cerebelli [11, 23, 24], make translation of pathology involving raised ICP and transtentorial herniation difficult. Following the poor clinical translation of promising treatments developed in rodent models, the STAIR guidelines [25] recommend that neuroprotective agents be tested in gyrencephalic species following initial rodent studies [8]. The neuroanatomical properties of the sheep [15], together with the physiological and histological outcomes we have demonstrated, suggest that our ovine MCAO model is suitable for pre-clinical testing after preliminary rodent studies in order to confirm efficacy prior to progression to clinical assessment.

Our ovine stroke model fulfills numerous STAIR criteria [25], allowing for extensive physiological monitoring including arterial blood pressure and blood gas analysis, multiple blood draws from the same animal at multiple time points, electrolyte and renal function, hemoglobin and hematocrit, fluid balance and core body temperature. An additional advantage is that it allows for reperfusion and we have investigated the effects of MCAO in female animals. Furthermore, we have demonstrated multiple outcome measures for proximal MCAO in the sheep, including physiological (ICP, PbtO_2_), radiological (DWI infarct volume, T2WI edema, midline shift and herniation syndromes) and pathological (TTC and immunohistopathology). We observed early mortality in 30% of sheep within 24 hours of permanent MCAO, secondary to cerebral herniation and brainstem compression, consistent with previous non-human primate models and large volume human stroke, making this model a powerful tool for investigating therapies targeting malignant cerebral edema and mortality. Although, such mortality in combination with the extensive extra-cranial dissection required to reach the proximal MCA, may be problematic for long-term survival studies.

### ICP

Our study is the only report of ICP after permanent and temporary MCAO in a gyrencephalic species for a clinically relevant minimum 24 hours. Several studies have described ICP after rodent stroke, however measurements tend to be taken from within the posterior fossa [13, 26]. Although due to the underdeveloped tentorium in the rat an infratentorial ICP monitor is probably an accurate reflection of supratentorial pressure following space-occupying cerebral edema. Nevertheless, the lack of significant compartmentalization between the cerebrum and cerebellum makes transtentorial herniation following rodent MCAO unlikely. Despite this, rodent MCAO is associated with space-occupying cerebral edema and midline shift [27]. In rodent stroke, decompression improves mortality rates and neurological function [28], suggesting that following large volume rodent MCA stroke ICP is still a contributing factor to outcome. Studies investigating ICP after ischemic stroke in gyrencephalic species include cats, dogs and baboons, however almost all have reported relatively short monitoring periods of only 6–12 hours post-stroke [29–31]. O’Brien and Waltz performed transorbital MCAO in cats and measured ICP for up to 15 days with technical difficulties [32]. ICP data from the present study differed slightly from our previous study where ICP was measured up to 4 hours following stroke onset. In our previous study, differences in ICP were not observed during the 4 hour monitoring period, whereas they were evident as early as 3 hours post-stroke onset in the present study. Possible explanations for these differences include surgical experience and improved technique, and the logarithmic exponential transformation with geometric mean data analysis used in the present study, both of which may result in more accurate data collection and analysis compared with the previous study.

Raised ICP is used frequently used as an outcome measure in clinical and experimental studies of traumatic brain injury but is less commonly in acute stroke, where elevated ICP is generally identified by radiological or clinical findings. Nevertheless, intracranial hypertension is a cause of early in-hospital mortality in ≤28.9% of stroke unit admissions (Koennecke et al, 2011). Hacke’s [3] original description of malignant MCA infarction included 26 patients with epidural ICP monitoring, assessing the efficacy of anti-edema therapy and barbiturate coma after radiological identification of space-occupying edema. Baseline ICP was 19.4mmHg, and over 4–10 days the average highest ICP recorded in patients that died was 43mmHg compared with 28mmHg in survivors. Ropper and Shafran [33] reported their ICP findings in 6 patients in an earlier series, and a larger series by Schwab suggested ICP>35mmHg was a poor prognostic sign [34]. However, the clinical usefulness of ICP monitoring for ischemic stroke is questionable at best and is not as useful an indicator to perform surgical decompression as clinical and neuroimaging findings [34]. However, in an experimental setting ICP monitoring is simple to perform and provides a quantifiable numerical value from which the physiological effectiveness of anti-space-occupying edema therapies can be assessed. Indeed, our post-stroke ICP monitoring revealed that our ovine MCAO model produced ICP elevations comparable to that observed clinically [3, 33, 34].

### PbtO_2_

We have previously characterized the regional PbtO_2_ response to permanent and temporary MCAO in the sheep, albeit only for 4 hours [15]. Nevertheless, we demonstrated reduced PbtO_2_ following temporary occlusion that returned to sham levels 30 minutes following reperfusion, and a sustained reduction in PbtO_2_ following permanent MCAO. The present study produced similar results but with some important differences. Firstly, it took 7 hours for PbtO_2_ in temporary MCAO to approach and become comparable with shams, more than 6 hours longer than our initial study. Secondly, temporary MCAO PbtO_2_ levels fell much lower in the present study, almost to permanent MCAO levels, and in fact declined faster than the permanent MCAO group. Possible explanations for these differences include surgical experience and improved technique, and the logarithmic exponential transformation with geometric mean data analysis used in the present study, both of which may result in more accurate data collection and analysis compared with the previous study. PbtO_2_ levels in sham animals were not entirely stable throughout the 24 hour monitoring period, this may be attributable to the fact that the probe can take several hours to equilibrate once inserted into the brain parenchyma and/or the influence of oxygen levels within the anesthesia gas delivery mix.

### MRI

The development of a malignant course following MCA stroke is frequently associated with large infarct volume, however inflammation and BBB breakdown may also be important parameters [35]. Large territory human MCA infarct volumes, such as DWI infarct volume >145cm^3^ [36] or Computerized Tomography ischemic changes affecting at least two-thirds of the MCA territory with basal ganglia involvement [37]) or space-occupying edema [38] are predictive of a malignant course and improved outcome following surgical decompression [6]. Indeed, our permanent MCAO findings are in keeping with such features of large MCA stroke in humans. Midline shift is a frequently associated, albeit relatively late finding, with the degree of midline shift correlating with edema volume in experimental rodent studies [27]. Compared with the smaller temporary MCAO infarcts, we found that permanent MCAO produced a significantly larger diffusion deficit and edema volume (approximately 25% of the supratentorial brain), as well as radiological evidence of raised ICP secondary to focal space-occupying pathology. Furthermore, permanent MCAO produced radiological evidence of raised ICP with larger DWI, T2WI and TTC volumes. Such findings on MRI are observed in the setting of clinical stroke and are a clinically relevant feature of our ovine model.

### Histopathology

Ischemic injury on H&E for both permanent and temporary MCAO mirrored TTC and DWI evidence of ischemia, and similarly the Weil stain (myelin degeneration) corresponded well with T2WI cerebral edema. TUNEL staining confirmed widespread cell loss throughout the infarcted hemisphere in permanent MCAO, compared to much smaller regions in the transient MCAO group, as reflected by the H&E and TTC staining. Albumin extravasation confirmed that disruption of the BBB and development of vasogenic edema is the main source of cerebral edema in this model. Indeed, albumin extravasation was significantly more prevalent following permanent MCAO compared with transient MCAO.

Multiple techniques have been described to study transient and permanent MCAO in animal models, with the effects of MCAO varying considerably depending upon the species, strain, method/duration of occlusion used. Here we describe a model of transient or permanent MCAO in the sheep. Transient occlusion of the MCA followed by reperfusion after 2 hours produced discrete, variable lesions characterized by small regions of BBB breakdown and cerebral edema, histopathology and MRI respectively. Transient MCAO in our ovine model produced a transient decline in PbtO_2_ levels that correlated with clip application and removal, with ICP remaining within normal limits during this time. This model permits the study of reperfusion injury associated with MCA occlusion and reperfusion. In contrast, permanent MCAO in our ovine model produced large infarcts encompassing the entire MCA territory, accompanied by widespread loss of BBB integrity, profound cerebral edema, significantly increased ICP, accompanied by a sustained decline in PbtO_2_ levels and a mortality rate of 30%. This permanent model is most appropriate for screening agents targeting cerebral edema and elevated ICP following stroke.

### Limitations

Although the findings suggest that this ovine model is a good candidate for pre-clinical screening of agents targeting cerebral edema and elevated ICP, the limitations of the study must be acknowledged. Firstly, institutional ethical approval was granted on the condition that animals remain under general anesthesia for the entire duration of the experiment, some 30 hours or so. We acknowledge that human patients are rarely anesthetized at the time of stroke, in addition to the potential neuroprotective effects of anesthesia on experimental stroke model outcomes. Indeed, many studies have now reported on the neuroprotective effects of anesthesia in animal models of stroke [39]. Specifically, ketamine is suggested to have neuroprotective properties through inhibition of apoptosis proteins and modulation of the inflammatory response [40]. Given that ketamine is a NMDA antagonist it may have influenced the extent of excitotoxic brain damage. However, ketamine has the specific advantage of possessing analgesic properties and although inhalational anesthetics are generally preferred for prolonged procedures, combination inhalational/ketamine regimes have been popular for and successful in anesthetizing small ruminants since the 1970s [41]. The addition of a low dose ketamine infusion to sub-neuroprotective MCA isoflurane produced a synergistic relationship between the two anesthetic agents, permitting as lower MCA isoflurane and therefore enabling good anesthesia and systemic blood pressure but avoiding inadvertent neuroprotection as a confounding factor when characterizing the stroke model. Nevertheless, the experiments were conducted within the constraints of the ethical approval requirements. Ideally, we would wake the animals up following the induction of stroke and track progress in awake animals. In conjunction with our ethics committees we are working to extend the model to permit long-term survival by modifying the surgical approach, thereby obviating the need for long-term anesthesia and allow the study of conscious animals following the induction of stroke. Furthermore, only female animals were used in the current study. Using both male and female animals would have been optimal but given the length of the experiment (~30 hours) with the animals under general anesthesia the entire time meant that a urinary catheter needed to be inserted. Females were selected preferentially over males upon veterinary advice given the difficulty in catheterizing the highly convoluted male urethra. Group sizes differed between stroke and sham groups. Group sizes did not differ intentionally and were a direct reflection of animal availability due to the age and weight range required. Only a subset of animals underwent MRI due to financial constraints and access to the scanner. Although animals were randomly selected and were not consecutive animals it would have been desirable to image each and every animal in the study.

In the present study ICP was measured in the contralateral hemisphere to avoid overcrowding in the ipsilateral hemisphere. There is some experimental evidence suggesting that a significant pressure gradient exists between hemispheres in gyrencephalic brains with unilateral space-occupying pathology including large volume stroke [29], whereas others believe pressure is transmitted uniformly within the supratentorial compartment [42]. A human case report suggests that gradients may only be apparent at the time of decompensation and intracranial shift [43]. Recording ICP in the right hemisphere may have yielded higher mean pressures, particularly after permanent MCAO, and bilateral recordings may have provided more information on interhemispheric pressure gradients following MCA stroke. Nevertheless, our contralateral recordings demonstrated a significant elevation in ICP following ischemic space-occupying cerebral edema. Finally, the differences in ICP response between the permanent and transient MCAO groups may have been influenced by the time to skull closure. In the permanent MCAO animals the dura/skull was closed immediately after vessel occlusion whereas in the transient group the dura/skull was left open for 2 hours during the occlusive period and only closed upon reperfusion. This may have influenced the time to restoration of intracranial dynamics, accumulation of CSF and ICP changes in response to the evolving infarct and any associated tissue swelling.

## Conclusions

We have demonstrated a malignant course following permanent proximal MCAO in an ovine model, characterized by elevated ICP, space-occupying cerebral edema, large diffusion deficit and a considerable mortality of 30% within 24 hours of stroke. Temporary MCAO with reperfusion produced smaller infarct volumes, localized cerebral edema without space-occupying effect or ICP alterations. This model provides a powerful tool for the preclinical investigation of novel therapies, particularly those targeting cerebral edema and elevated ICP. This model may be used to supplement the currently used small animals models to validate agents of outstanding promise prior to clinical assessment. We believe this step-wise progression of screening through small animal models then large animal models will increase the likelihood of clinical success and improve translation of stroke therapeutics.
